# Cytotaxonomy of two species of genus *Chrysolaena* H. Robinson, 1988 (Vernonieae, Asteraceae) from Northeast Paraguay

**DOI:** 10.3897/CompCytogen.v8i2.7209

**Published:** 2014-05-19

**Authors:** Gisela M. Via Do Pico, Massimiliano Dematteis

**Affiliations:** 1 Instituto de Botánica del Nordeste (UNNE-CONICET), Sargento Cabral 2131. Casilla de Correo 209, CP 3400 Corrientes, Argentina

**Keywords:** B chromosomes, chromosome numbers, karyotype, Lepidaploinae, polyploidy

## Abstract

Chromosome counts and karyotypes of two species of *Chrysolaena* H. Robinson 1988 are presented in this paper. Mitotic analysis revealed that both taxa have *x*=10, a basic chromosome number considered characteristic of the genus. The chromosome number and the karyotype of *Chrysolaena cristobaliana* are reported for the first time, as well as a new cytotype and the karyotype of *Chrysolaena sceptrum*. *Chrysolaena cristobaliana* showed heptaploid cytotype with 2*n*=7*x*=70 and a karyotype composed of 46 *m* + 24 *sm* chromosomes. On the other hand, *Chrysolaena sceptrum* presented tetraploid cytotype with 2*n*=4*x*=40 and a karyotype with 30 *m* + 10 *sm* chromosomes. Accessory chromosomes were observed in cells of both species. The chromosome analysis showed that these species differ in the chromosome number and the total chromosome length, although they showed similar chromosome morphology and asymmetry indexes. The results support the use of chromosome data in taxonomic treatments of the American members of the tribe Vernonieae.

## Introduction

The genus *Chrysolaena* (Vernonieae, Asteraceae) includes 18 species mainly concentrated in southern Brazil and northeast of Argentina. From this area, the genus extends to north Peru and the Amazon region of Brazil, and southward, to the center of the province of Buenos Aires in Argentina. Most of the species of *Chrysolaena* occur in Brazil (15 spp.), Paraguay (11 spp.) and Argentina (6 spp.). However, a small number of species are found in Bolivia, Peru and Uruguay. Species of the genus are characterized by sericeous or velutinous indumentum, glandular anthers appendages, style without a basal node and glandular cypselas (Robinson 1988). Another important distinguishing feature that separates it from the other American genera of the tribe is the morphology of the pollen grains (Type C) which is tricolporate, echinolophate, with presence of polar lacuna but lacking of equatorial lacuna (Keeley and Jones 1979, Via do Pico and Dematteis 2013). However, the more distinguishable feature of *Chrysolaena* is probably the base chromosome number, because this is the single American genus of the tribe with *x*=10 (Dematteis 1997, Via do Pico and Dematteis 2012a, 2012b).

Since the taxonomic treatment realized by Robinson (1988), where the author segregates *Chrysolaena* from *Vernonia* Schreb., 1791, most of the studies have been focused in nomenclature, anatomy, cytology and palynology (Martins and Oliveira 2007, Oliveira et al. 2007b, Mendonça et al. 2007, Dematteis 2009, Galastri et al. 2010, Via do Pico and Dematteis 2012a, 2012b, 2013, Appezzato-da-Glória et al. 2012). Despite these contributions, the chromosome information is still scarce. Chromosome studies carried out in the genus, reported basic number *x*=10 and different ploidy levels or cytotypes in nine species of *Chrysolaena*: both diploid and tetraploid populations have been found in *Chrysolaena flexuosa* (Sims) H. Robinson, 1988, *Chrysolaena propinqua* (Hieron.) H. Robinson, 1988, *Chrysolaena lithospermifolia* (Hieron.) H. Robinson, 1988, and *Chrysolaena obovata* (Less.) Dematt., 2009, whereas only diploid populations are known for *Chrysolaena verbascifolia* (Less.) H. Rob., 1988. In *Chrysolaena simplex* (Less.) Dematt., 2007, have been found tetraploid cytotypes and in *Chrysolaena sceptrum* (Chodat) Dematt., 2009, octoploid. Both *Chrysolaena cognata* (Less.) Dematt., 2009, and *Chrysolaena platensis* (Spreng.) H. Robinson, 1988, show a greater cytological variation with diploid, tetraploid, hexaploid, and octoploid populations, and even odd polyploids in *Chrysolaena cognata* (Galiano and Hunziker 1987, Dematteis 1997a, 2002, 2009, Angulo and Dematteis 2009b). Despite these studies, only the karyotypes of *Chrysolaena flexuosa*, *Chrysolaena simplex*, *Chrysolaena platensis*, *Chrysolaena cognata*, *Chrysolaena verbascifolia*, *Chrysolaena propinqua* and *Chrysolaena lithospermifolia* have been analyzed (Ruas et al. 1991, Dematteis 1997a, Angulo and Dematteis 2009a, 2009b, Via do Pico and Dematteis 2012b) and these analyses did not include all the cytotypes of the species.

*Chrysolaena cristobaliana* Dematt., 2009, and *Chrysolaena sceptrum* are erect shrubs with well-developed xylopodia and its distribution is mostly restricted regarding to the other species of the genus. Both taxa grow on high fields and “Cerrados” from northeast of Paraguay and southeastern Mato Grosso and Mato Grosso do Sul in Brazil (Dematteis 2009). Cytological information of these two species is very scarce and only the chromosome number of a single population of *Chrysolaena sceptrum* has been reported (Dematteis 2002).

In the present study, *Chrysolaena cristobaliana* and *Chrysolaena sceptrum* were cytologically examined in order to extend the cytogenetic knowledge and provide information taxonomically useful. The chromosome number and the karyotype of *Chrysolaena cristobaliana* are reported for the first time, as well as a new cytotype and the karyotype of *Chrysolaena sceptrum*.

## Materials and methods

The specimens were obtained from natural populations from department of Amambay, northeast of Paraguay. Voucher specimens are kept at the herbarium of the Instituto de Botánica del Nordeste (CTES). Location and Voucher specimens: *Chrysolaena cristobaliana*: Paraguay, Dpto. Amambay: Chirigüelo, 2 km W Pedro Juan Caballero. Cerrado degraded, near neighborhood. *Dematteis and Vega 4283*, (CTES). *Chrysolaena sceptrum*: Paraguay, Dpto. Amambay: Chirigüelo, 2 km W Pedro Juan Caballero. Cerrado degraded, near neighborhood. *Dematteis and Vega*, *4289* (CTES).

Mitotic chromosome preparations were made from root meristems obtained from germinating seeds. The roots were pretreated for about 5 h in 0.002 M 8-hydroxyquinoline solution at room temperature, fixed in 3:1 absolute alcohol/acetic acid, and then stained using Feulgen’s technique. Permanent microscope slides were prepared by mounting in Euparal.

At least 10 metaphases were drawn for each population using a Zeiss camera lucida (Carl Zeiss, Germany), selecting the best for measurements. The nomenclature used to describe the chromosome morphology was the one proposed by Levan et al. (1964). The morphology of the chromosomes was determined using the centromeric index (i=short arm x 100/total length of the chromosome). Accordingly, the chromosomes were classified as metacentrics (*m*): 50–37.5, submetacentrics (*sm*): 37.5–25, and subtelocentrics (*st*): 25–12.5. Ideograms were drawn based on the average centromeric index and arranged in order of decreasing size. Because the polyploid nature of the species is unknown and taking into account the concept of ideogram (diagrammatic representation of the gametic chromosome set (n) of a species), the chromosomes were grouped in pairs.

The following karyological parameters were evaluated: total karyotype length (TKL), centromeric index (i), chromosome length (c), arm ratio (ar), and their averages (I, C and AR, respectively); in addition, the ratio between the smallest and the largest chromosome (R</>) was calculated. The karyotype asymmetry was estimated using intrachromosomal (A_1_) and interchromosomal (A_2_) indexes suggested by Romero Zarco (1986) and the symmetry classes of Stebbins (SC) (Stebbins 1971).

## Results

The species analyzed, the somatic chromosome numbers, the ploidy level, and the karyotypic parameters calculated are detailed in Table 1.

**Table 1.** Chromosomal number, ploidy level, karyotype formula, total karyotype length (TKL), average chromosome length (C), average centromeric index (I), average arm ratio (AR), ratio between the smallest and the largest chromosome (R</>**),** intrachromosomal asymmetry index (A_1_) and interchromosomal asymmetry index (A_2_), symmetry classes of Stebbins (SC) of the *Chrysolaena* species analyzed. SE: standard error.

**Species and voucher**	***Chrysolaena cristobaliana*** **4283**	***Chrysolaena sceptrum* 4289**
**2n**	70	40
**Ploidy**	7x	4x
**Karyotype formula**	2n=46m+24sm+0-7Bs	2n=30m+10sm+0-2Bs
**TKL ± SE (µm)**	92.42 ± 0.10	41.56 ± 0.09
**C (µm)**	2.64	2.08
**I ± SE**	39.71 ± 0.65	40.61 ± 0.85
**AR ± SE**	0.66±0.019	0.69±0.024
**R </>**	0.43	0.48
**A_1_**	0.33	0.31
**A_2_**	0.23	0.21
**SC**	1B	1B

Both species analyzed presented base chromosome number *x*=10. *Chrysolaena sceptrum* showed a tetraploid cytotype with 2n=4*x*=40 (Fig. 1a, b), with a karyotype composed of 30 metacentric (*m*) and 10 submetacentric (*sm*) chromosomes (Figure 2a). In a few cells from 0-2 accessory or B chromosomes were observed. These elements were metacentric and showed an average size of 1.47 µm. *Chrysolaena cristobaliana* presented a heptaploid cytotype with 2*n*=7*x*=70, 0-7 accessory chromosomes per cell (Fig. 1c, d) and a karyotype formed by 46 (*m*) and 24 (*sm*) chromosomes (Fig. 2b). The accessory chromosomes displayed a metacentric morphology, and an average size of 1.61 µm. A single secondary constriction was observed in pair N° 25.

**Figure:**
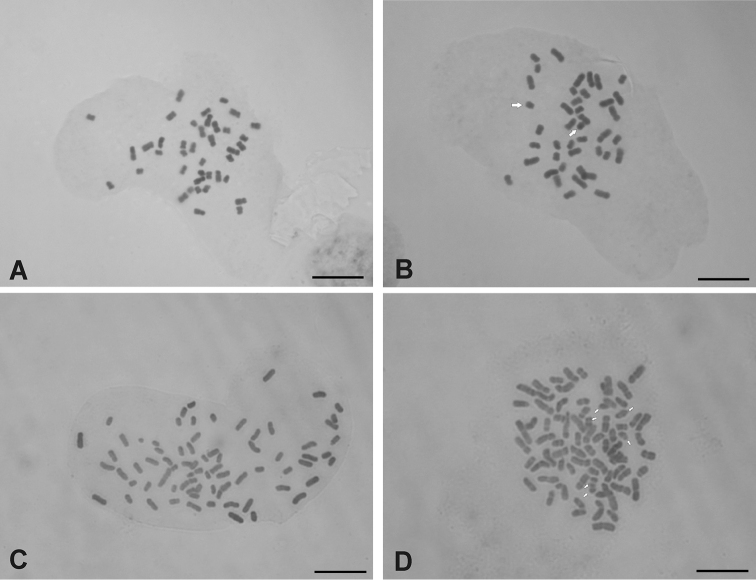
**Figure 1. A–D** Somatic chromosomes of *Chrysolaena*. **A–B** *Chrysolaena sceptrum*: **A** 2*n*=4*x*=40 **B** 2*n*=4*x*=40+2 Bs **C–D** *Chrysolaena cristobaliana*: **C** 2*n*=7*x*=70 **D** 2*n*=7*x*=70+6 Bs. Bar= 5 µm. White arrows denote B-chromosomes.

**Figure:**
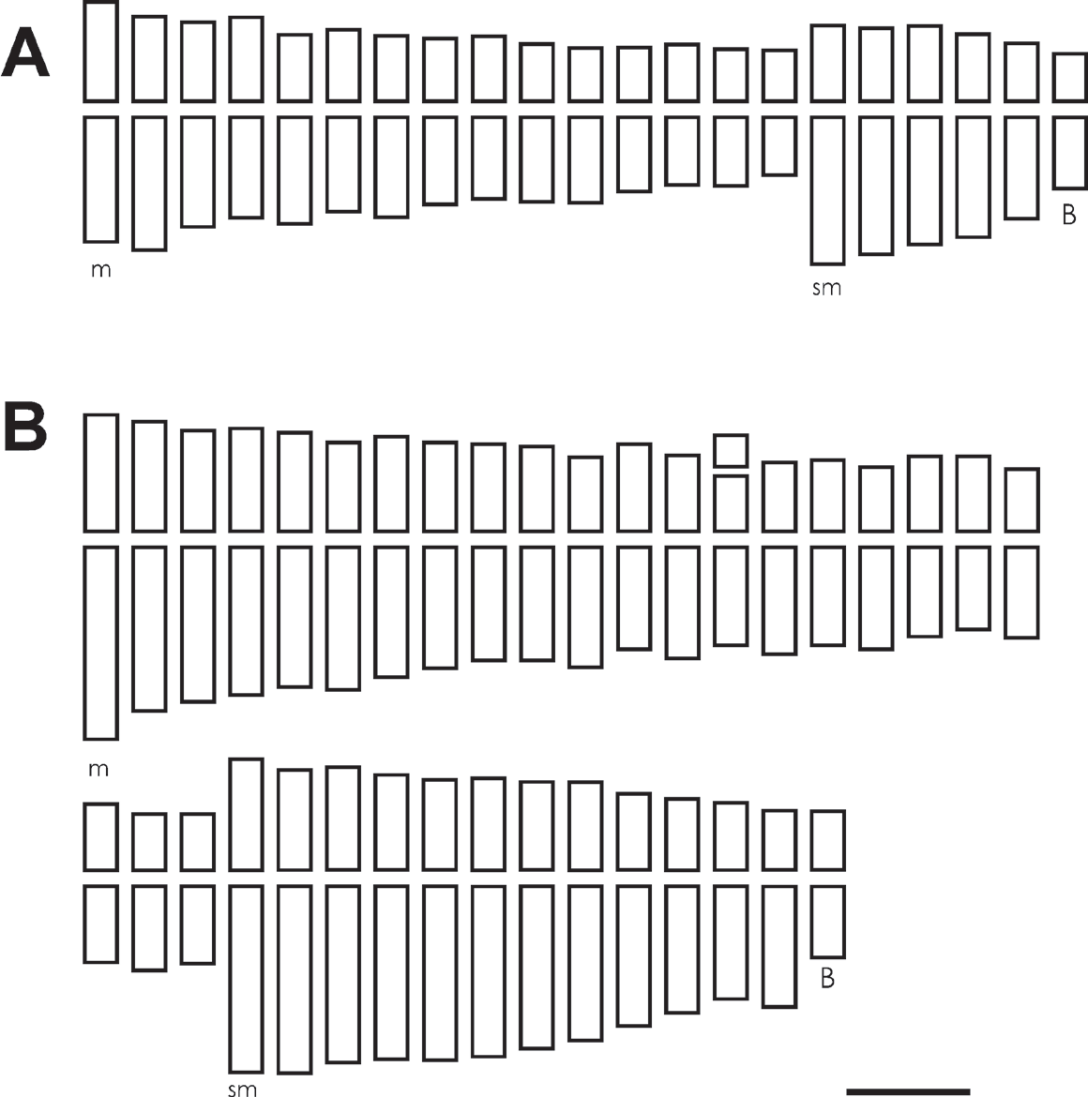
**Figure 2. A–B** Ideograms. **A** *Chrysolaena sceptrum*: 2*n*=30*m*+10*sm*+0-2 Bs **B** *Chrysolaena cristobaliana*: 2*n*=46*m*+24*sm*+0-7 Bs. Bar= 1.5 µm.

Both taxa showed moderately symmetrical karyotypes. The majority of chromosomes were metacentric, with fewer submetacentric pairs. Gradual differences in chromosome size were observed. *Chrysolaena cristobaliana* showed an average centromeric index I=39.71, an asymmetry index A_1_=0.33 and A_2_=0.23; while *Chrysolaena sceptrum* presented values of I= 40.61, A_1_= 0.31 and A_2_= 0.21. The average chromosome length (C) in *Chrysolaena cristobaliana* was 2.64 µm, while in *Chrysolaena sceptrum* it was 2.08 µm (see Table 1). According to the classification of Stebbins (1971) both species presented symmetry type 1B.

## Discussion

### Chromosome numbers

In this study we reported for the first time the chromosome number and the karyotype of *Chrysolaena cristobaliana*. Besides, the karyotype of *Chrysolaena sceptrum* was recorded for the first time, as well as a new cytotype.

The base chromosome number *x*=10 is considered characteristic of *Chrysolaena* and clearly distinguishes this genus from the remaining American groups of the tribe. This number also has been found in the two species here analyzed, which is consistent with previous studies carried out in others *Chrysolaena* species (Dematteis 1997a, 1997b, 1998, 2002, 2009, Dematteis et al. 2007, Angulo and Dematteis 2009a, 2009b, Via do Pico and Dematteis 2012a, 2012b). The only cytological record available for *Chrysolaena sceptrum* was reported by Dematteis (2002) for a population from Paraguay, which showed an octoploid cytotype with 2*n*=8*x*=80. In our study we report a new cytotype (tetraploid, 2*n*=4*x*=40) and the first karyotype analysis of the species. *Chrysolaena sceptrum* has been considered by some authors as a synonym of *Chrysolaena cognata* (Jones 1981, Robinson 1988), or a variety of this species (Cabrera 1944). However, there are many differences between these two entities, such as the shape and width of the leaves, and the number of florets per head, among the most conspicuous features (Dematteis 2009). Dematteis (2009) refers to the number of chromosomes as another distinguishing characteristic between these two species. *Chrysolaena cognata* presents 2*n*=20, 40, and 60, and *Chrysolaena sceptrum* presents octoploid cytotype (2*n*=80). Nevertheless, further counts reported also the octoploid cytotype (2*n*=80) for *Chrysolaena cognata* (Via do Pico and Dematteis 2012b), and in this study we report the tetraploid cytotype (2*n*=40) for *Chrysolaena sceptrum*. For this reason, the number of chromosomes already would not be a useful character to distinguish the two species each other. Although *Chrysolaena sceptrum* has been cited for Argentina (Cabrera 1944), it has not been found to date in that country. The specimens cited by Cabrera (1944) clearly belong to *Chrysolaena cognata*, which is widely distributed in Argentina. The geographic distribution of *Chrysolaena sceptrum* is exclusive of Mato Grosso and Mato Grosso do Sul in Brazil and east of Paraguay. Morphological differences listed above and geographical distribution would be the most conspicuous features to distinguish between *Chrysolaena cognata* and *Chrysolaena sceptrum*.

*Chrysolaena cristobaliana* has never been cytologically analyzed. This study reports as novelty the base chromosome number (*x*=10), the chromosome number (2*n*=7*x*=70, heptaploid) and the karyotype of the species. *Chrysolaena cristobaliana* and *Chrysolaena sceptrum* are closely related species. They are distributed in the same geographic region, and even populations of both entities can be found living in the same area. The main morphological features that differentiate these two species are the branch of the stem and the leaf shape. *Chrysolaena cristobaliana* presents densely branched and elliptical, lanceolate to oblanceolate leaves, whilst *Chrysolaena sceptrum* has single stems and narrowly lanceolate to linear leaves. The chromosome counts realized here show that these two entities can also be distinguished by the chromosome number.

*Chrysolaena cristobaliana* is also closely related to *Chrysolaena cognata*, one of the most widely distributed species of the genus. However, both species differ in the leaf shape, the pubescence type, the florets number and the geographical distribution. The results of this study added the chromosome number as a feature to distinguish these two closely related species.

### B chromosomes

Jones (1995) estimated that accessory chromosomes occur in about 10–15% of flowering plant species. Their distribution among angiosperm families is quite heterogeneous. They have been described in more than 150 species of Asteraceae and Poaceae. Generally, these chromosomes are heterochromatic, small, and very variable in number between individuals (Guerra 1988). The irregular distribution of B chromosomes among Angiosperm families suggests that species in certain groups are more likely to bear them than species in other families (Levin et al. 2005). Numerous cases of accessory chromosomes are known in the tribe Vernonieae (Angulo and Dematteis 2009b, Dematteis 1998, Galiano and Hunziker 1987, Oliveira et al. 2007a, Angulo and Dematteis 2012). The occurrence of accessory chromosomes in *Chrysolaena cristobaliana* and *Chrysolaena sceptrum* are new records for the genus. In both species, Bs presented a metacentric morphology and similar size. Previous studies reported accessory chromosomes in *Chrysolaena flexuosa*, *Chrysolaena cognata*, *Chrysolaena propinqua*, and *Chrysolaena verbascifolia* (Via do Pico and Dematteis 2012b). In most of this species, Bs are present in a low frequency (1-4), as reported here in *Chrysolaena sceptrum*. However, in *Chrysolaena verbascifolia* it has been observed between 0 and 7 B chromosomes per cell, as well as in *Chrysolaena cristobaliana*. This variation was observed between individuals of the same population and even within the same individual. Another case in which it has been observed a high frequency of B chromosomes is *Lepidaploa canescens* (Kunth) H. Robinson, 1990, (sub nom=*Vernonia geminata*). In this species it has been observed from 0 to 6 accessory chromosomes amongst the cells of a given individual (Oliveira et al. 2007a). The B chromosomes of *Chrysolaena cristobaliana* and *Chrysolaena sceptrum* varied in cells of the same plant, which suggests non disjunction in the mitotic anaphase. Variation in chromosome number in the same plant is a rule used to discriminate B chromosomes from the normal chromosome complement (Jones and Rees 1982). Apparently, there is no difference in frequency between diploid and polyploids species (Jones and Rees 1982, Palestis et al. 2004, Trivers et al. 2004), but, there is a trend suggesting that Bs have a higher frequency in species with a large genome size (Trivers et al. 2004). So far, in the analyzed species of *Chrysolaena*, there is no differences in frequency between diploids and polyploids, since in *Chrysolaena verbascifolia* (2*n*=20) and *Chrysolaena cristobaliana* (2*n*=70) it has been observed the same frequency of Bs.

In species of plants and animals, that carries B chromosomes, those individuals from a given population with and without Bs, cannot generally be phenotypically distinguished from each other. However, in some species, there are instances in which B chromosomes change certain morphological characteristics (Jones and Rees 1982, Jones and Houben 2003) or cause some selectively advantageous effects (Teoh and Jones 1978, Jones and Rees 1982). Apparently, the B chromosomes found in *Chrysolaena* species have no effect on the phenotype or development of the individuals.

### Karyotype

*Chrysolaena cristobaliana* and *Chrysolaena sceptrum* were never been karyotypically characterized. Karyotype analysis is essential for the cytogenetic characterization of species and to examine the variation between its individuals and/or populations. The comparison of karyotypes of different species also allows the taxonomic and evolutionary analysis of a taxon, such as a genus. Besides, many times, differences in karyotype asymmetry can indicate how these chromosomes have diversified in size and morphology within a group (Guerra 1988). The karyotypes of the taxa here studied are formed by metacentric and submetacentric chromosomes, and are quite symmetrical due the predominance of metacentric chromosomes. This is a common feature in species of the tribe Vernonieae (Ruas et al. 1991, Dematteis 1997a, 1997b, 1998, Dematteis and Fernández 1998, 2000, Oliveira et al. 2007a, 2007b, Angulo and Dematteis 2009b, Via do Pico and Dematteis 2012b). *Chrysolaena sceptrum* presents the most symmetric karyotype, which is reflected in the highest average centromeric index and the lowest intrachromosomal asymmetry coefficient (A_1_). *Chrysolaena cristobaliana* shows the highest interchromosomal asymmetry coefficient (A_2_), which reflects the amplitude of its chromosome size. The asymmetry indexes, A_1_ and A_2_, calculated show similar values to other species of *Chrysolaena* previously analyzed (Angulo and Dematteis 2009b, Via do Pico and Dematteis 2012b). According to Stebbins (1971), both species fit into the symmetry category 1B, since the relation between the longest and shortest chromosome was between 2:1 - 4:1. This author suggested that classes 1B and 1C are absent in higher plants and only occur in animals (particularly in reptiles), which have karyotypes characterized by great differences in chromosome size, but with predominantly median or submedian centromeres (Stebbins, 1971). However, several subsequent works demonstrated that the class 1B is present among plants, e.g. in the genera *Helianthus* Linnaeus, 1753, *Crotalaria* Linnaeus, 1753, and *Onobrychis* Miller, 1754 (Gupta and Gupta 1978; Kulshreshtha and Gupta 1981; Almada et al. 2006; Hesamzadeh Hejazi and Mahdi Ziaei Nasab 2010).

*Chrysolaena cristobaliana* presents the longest karyotype (TKL), which is correlated with its ploidy level (2*n*=7*x*=70). Although the species differed in their chromosome number and total karyotype length, they had similar chromosomal morphology and asymmetry indices. Therefore, karyotype data do not seem to be of great use for group taxonomy, since the chromosomes are small and the karyotype differences cannot be detected rapidly by the analysis of one or a few cells of each species, but only by comparing average measurements. According to Ruas et al. (1991), Dematteis (1996, 1998), Dematteis and Fernández (1998, 2000) and Oliveira et al. (2007a, b), despite the occurrence of variation in chromosome number among species of *Vernonia sensu lato*, the karyotypes with conventional techniques does not discriminate well the species already studied, due to small variation in chromosome size and centromeric position.

### Ploidy levels

The species of *Chrysolaen*a exhibit abundant polyploidy. The majority of species studied so far, include diploid and polyploid populations. There are some exceptions of species with a single known cytotype, such as the diploid *Chrysolaena verbascifolia* (Dematteis 1997, Via do Pico and Dematteis 2012b). The review of chromosomal studies reveals that the tetraploid cytotype is the most common. From nine species in which chromosome number is known, seven had populations with tetraploid cytotypes (Dematteis 1997a, 1997b, 1998, 2002, 2009, Dematteis et al. 2007, Angulo and Dematteis 2009b, Via do Pico and Dematteis 2012a, b). Ploidy differences are not restricted to comparisons between species, but also occur frequently within species (Miller 1978, Burton and Husband 1999, Weiss et al. 2003). Available data on *Chrysolaena* and the results of this study show that the genus is cytologically complex, and polyploidy is an important mechanism in the differentiation and adaptation of species. The species, as well as, different populations of same species present numerous ploidy levels, even odd cytotypes. Some species would be polyploid series and would present more than one cytotype. Besides, there may be a species complex, with entities morphologically related but with different ploidy levels. In this group of species it could be occurring continuous processes of polyploidization and hybridization, which would lead to the different cytotypes observed. According to Jones (1979) and Ruas et al. (1991), the Vernonieae of the New World, in contrast to those of the Old World, show marked diversity in chromosome number and a high ratio of polyploid species. Polyploids may combine to give rise to a complex of polyploid species, which promotes morphological and ecological changes that hinder the taxonomic treatments (Galiano and Hunziker 1987, Dematteis 2002).

Moreover, the chromosome number found in *Chrysolaena cirstobaliana* is the second report of impair ploidy level for the genus *Chrysolaena*. In *Chrysolaena cognata* a mixed population with pentaploid (2*n*=5*x*=50) and hexaploid specimens (2*n*=6*x*=60) was found in Misiones, Argentina (Dematteis 2002). However, the population of *Chrysolaena cristobaliana* is not mixed, and all individuals analyzed showed heptaploid ploidy level (2*n*=7*x*=70). Within the Vernonieae, another case of odd polyploidy was reported in *Lessingianthus macrocephalus* (Less.) H. Robinson, 1988, which presented 2*n*=11*x*=176, one of the higher ploidy levels found within the Asteraceae (Angulo and Dematteis 2012). The high ploidy level with odd chromosome complement, suggests that an irregular meiosis behavior could lead to sterility. Generally, in plants of the Asteraceae family, with these features, is very common the apomictic reproduction. Apomixis is usually defined as a natural process that allows clonal reproduction through seeds, avoiding meiosis and fertilization, and resulting in offspring that are genetically identical to the maternal plant (Nogler 1984). Apomixis was recorded in 2.9 % of the genera of the Asteraceae family and is very common, e.g., in the tribe Eupatorieae (Farco et al. 2012). Considering this background, the population of *Chrysolaena cristobaliana* analyzed in this study probably present apomictic reproduction. However, other studies should be conducted to test this issue.

## Conclusions

There are many important characters of taxonomic weight to separate *Chrysolaen*a from other Vernonieae groups, such as the morphology of the pollen grains and the morphological characters. However, the chromosome number is considered one of the most important features, since *Chrysolaena* is the only American member of the tribe with the base number *x*=10, which is mainly present in the Old World Vernonieae. Historically, base chromosome numbers have been widely employed for the delimitation of generic and infrageneric taxa in the Compositae (Sundberg et al. 1986). Studies from pollen morphology previously carried out in *Chrysolaena* showed the occurrence of pollen type C (proposed as typical of the genus) in *Chrysolaena cristobaliana* and *Chrysolaena sceptrum* (Via do Pico and Dematteis 2013). In addition to this, the chromosome counts carried out in these two species confirm the base chromosome number of *x*=10 proposed for the genus, and support the taxonomic position of these entities. These results contribute to the knowledge regarding the cytology of the *Chrysolaena* genus and support the use of chromosome number for the taxonomy of the American Vernonieae.
